# Epidemiologic and Clinical Features of Mpox in Transgender and Gender-Diverse Adults — United States, May–November 2022

**DOI:** 10.15585/mmwr.mm715152a1

**Published:** 2022-12-30

**Authors:** Dawn Blackburn, Nicole M. Roth, Jeremy A.W. Gold, Leah Zilversmit Pao, Evelyn Olansky, Elizabeth A. Torrone, R. Paul McClung, Sascha R. Ellington, Kevin P. Delaney, Neal Carnes, Patrick Dawson

**Affiliations:** ^1^Epidemic Intelligence Service, CDC; ^2^CDC Mpox Emergency Response Team; ^3^Division of HIV Prevention, National Center for HIV, Viral Hepatitis, STD, and TB Prevention, CDC.

As of November 9, 2022, a total of 28,730 cases of monkeypox (mpox) had been reported in the United States,* primarily among adult cisgender men reporting recent male-to-male sexual contact (*1*). Transgender and gender-diverse persons, who constitute an estimated 0.5% of the U.S. adult population,^†^ face unique health disparities and barriers to care (*2*–*4*). However, data on the epidemiologic and clinical features of *Monkeypox virus* infections in this population are limited (*5*). CDC analyzed U.S. case surveillance data on mpox cases in transgender and gender-diverse adults reported during May 17–November 4, 2022. During this period, 466 mpox cases in transgender and gender-diverse adults were reported, accounting for 1.7% of reported cases among adults. Most were in transgender women (43.1%) or gender-diverse persons (42.1%); 14.8% were in transgender men. Among 374 (80.3%) mpox cases in transgender and gender-diverse adults with information available on sexual or close intimate contact, 276 (73.8%) reported sexual or close intimate contact with a cisgender male partner during the 3 weeks preceding symptom onset. During the ongoing outbreak, transgender and gender-diverse persons have been disproportionately affected by mpox. Members of this population frequently reported recent sexual or close intimate contact with cisgender men, who might be in sexual networks experiencing the highest incidence of mpox. These findings highlight the importance of tailoring public health prevention and outreach efforts to transgender and gender-diverse communities and could guide strategies to reduce mpox transmission.

Data on confirmed and probable cases of mpox are electronically reported by jurisdictional health departments to CDC using a standardized case report form^§^ or the National Notifiable Diseases Surveillance System.^¶^ CDC analyzed case report form data for persons aged ≥18 years with probable or confirmed mpox reported through November 4, 2022. CDC identified persons as transgender or gender-diverse if their self-reported gender** was transgender or “another gender identity” (i.e., not cisgender or transgender); in addition, persons whose self-reported gender identity differed from their assigned sex at birth were considered transgender.^††^ This descriptive analysis included demographic and epidemiologic characteristics, exposure characteristics, symptoms, HIV status, and hospitalization status. Data were stratified by gender identity. Because of the high level of missingness of some variables, statistical testing was not performed. This activity was reviewed by CDC and was conducted consistent with applicable federal law and CDC policy.^§§^

As of November 4, 2022, a total of 28,072 cases of mpox had been reported in U.S. adults, primarily among cisgender men (94.8%); 2.6% of cases occurred in cisgender women (Table 1). A total of 466 (1.7%) adults with mpox were transgender or gender-diverse; among these persons, most were transgender women (43.1%) or gender-diverse persons (42.1%); 14.8% were transgender men. A total of 223 persons with missing age were excluded. Among 157 (80.1%) cases in gender-diverse adults with available data on assigned sex at birth, 151 (96.2%) were assigned male sex at birth.

**TABLE 1:** Gender identity* of adults who received a diagnosis of mpox (N = 28,072) — United States, May–November 2022

Gender identity	No. (%)
**Cisgender, total**	**27,352 (97.4)**
Cisgender men	26,616 (94.8)
Cisgender women	736 (2.6)
**Transgender and gender-diverse, total**	**466 (1.7)**
Gender-diverse persons^†^	196 (0.7)
Transgender women	201 (0.7)
Transgender men	69 (0.2)
Missing	254 (0.9)
**All persons**	**28,072 (100)**

* Because of differences in collection of sex and gender information among jurisdictions, both sex and gender were used to represent gender identity. Persons whose reported sex differed from their reported gender were classified as transgender (59). Self-reported gender or sex assigned at birth were missing for 10,370 (36.9%) adults and their gender identity was presumed to be cisgender consistent with the available response unless their gender identity was reported as transgender or “another gender identity.”

^†^ Persons whose self-reported gender was “another gender identity” (i.e., not transgender or cisgender), such as nonbinary, genderqueer, and gender nonconforming, were classified as gender-diverse.

Overall, approximately 52.1% of cases in transgender and gender-diverse adults were reported from New York City (26.0%) or California (26.2%). The median age of transgender and gender-diverse adults with mpox was 32 years (range = 18–71 years) (Table 2). Among the 416 (89.3%) transgender and gender-diverse adults with mpox for whom race and ethnicity were reported, 37.0% of cases occurred in Hispanic or Latino (Hispanic)^¶¶^ persons, 28.1% in non-Hispanic White persons, 27.6% in non-Hispanic Black or African American (Black) persons, and the remainder in persons of another race or ethnicity. The racial and ethnic distribution among transgender and gender-diverse persons with mpox was generally similar to that among cisgender persons.

**TABLE 2:** Demographic, epidemiologic, and clinical features of transgender, gender-diverse, and cisgender adults who received a diagnosis of mpox — United States, May–November 2022

Characteristic	No. (%)*	
	Transgender and gender-diverse persons (n = 466)	Cisgender persons (n = 27,352)
**Median age, yrs (range)**	32 (18–71)	34 (18–89)
**Race and ethnicity^†^**		
American Indian or Alaska Native	2 (0.5)	104 (0.5)
Asian	11 (2.6)	691 (3.0)
Black or African American	115 (27.6)	7,417 (32.2)
Hispanic or Latino	154 (37.0)	7,132 (30.9)
Native Hawaiian or other Pacific Islander	1 (0.2)	65 (0.3)
White	117 (28.1)	6,939 (30.1)
Multiracial or other race or ethnicity	16 (3.8)	716 (3.1)
Missing	50 (—)	4,288 (—)
**Sex or close intimate contact in the 3 wks before symptom onset**		
Any recently reported sexual or close intimate contact	316 (84.5)	13,556 (82.1)
Recent partners exclusively cisgender men	261 (69.8)	11,610 (70.3)
Recent partners include cisgender men and other genders	15 (4.0)	418 (2.5)
Recent partners exclude cisgender men	10 (2.7)	752 (4.6)
Genders of all partners unknown or not specified	30 (8.0)	776 (4.7)
No recently reported sexual or close intimate contact	58 (15.5)	2,962 (17.9)
Missing	92 (—)	10,834 (—)
**HIV infection status**		
HIV positive	79 (47.6)	3,469 (55.1)
HIV negative	87 (52.4)	2,825 (44.9)
Missing	300 (—)	21,058 (—)
**Hospitalized**		
Yes^§^	21 (6.9)	800 (6.5)
No	284 (92.8)	11,574 (93.5)
Missing	160 (—)	14,978 (—)

* Percentages were calculated using nonmissing data.

^†^ Persons of Hispanic or Latino (Hispanic) origin might be of any race but are categorized as Hispanic; all racial groups are non-Hispanic.

^§^ Among the 21 transgender and gender-diverse persons who were hospitalized, reason for hospitalization was available for 11 (52%): five were hospitalized for pain control, two for treatment of secondary infections, two for exacerbation of an underlying condition, and two for breathing problems, one of whom required mechanical ventilation; five were hospitalized for other reasons (patients could be hospitalized for more than one reason). HIV status was available for 18 (86%) of the hospitalized transgender and gender-diverse persons, among whom 13 had HIV infection.

Among 374 (80.3%) transgender and gender-diverse adults with sexual and close intimate contact information available, 316 (84.5%) reported engaging in any sexual or close intimate contact during the 3 weeks preceding symptom onset, including 276 (73.8%) who reported sexual or close intimate contact with a cisgender man (261 had exclusively cisgender men as partners, and 15 had partners who included cisgender men and persons of other genders). Ten (2.7%) transgender and gender-diverse adults with mpox reported exclusive sexual or close intimate contact with partners who were not cisgender men. Similarly, among 16,518 (60.4%) cisgender adults with this information available, 13,556 (82.1%) reported engaging in sex or close intimate contact during the 3 weeks before symptom onset, most often with a cisgender man.

The most frequently reported signs and symptoms reported by transgender and gender-diverse adults with mpox included rash (91.8%), malaise (67.3%), fever (64.9%), pruritis (63.9%), headache (63.7%), chills (62.2%) myalgia (61.9%), and enlarged lymph nodes (58.9%). Among 166 (35.6%) transgender and gender-diverse adult mpox patients with data available on HIV status, 79 (47.6%) had HIV infection, including 34 of 57 (59.6%) transgender women, six of 21 (28.6%) transgender men, and 39 of 88 (44.3%) gender-diverse persons. HIV prevalence among cisgender adults with mpox with available data was 55.1%. Among 306 transgender and gender-diverse adults with hospitalization data, 21 (6.9%) were hospitalized, similar to 6.5% of cisgender adults with available data. Among the 21 transgender and gender-diverse persons who were hospitalized, nine (43%) were transgender women, seven (33%) were gender-diverse persons, and five (24%) were transgender men. To date, no mpox-associated deaths have been reported among the transgender or gender-diverse adults identified in this analysis.

## Discussion

Mpox cases among transgender and gender-diverse adults have accounted for 1.7% of total U.S. cases. Based on the estimated percentage of U.S. adults who identify as transgender or gender-diverse (0.5%), this population has been overrepresented among mpox cases during the ongoing outbreak (*2*). Among transgender and gender-diverse persons with available sexual and close intimate contact information, a commonly reported potential exposure was recent sexual or close intimate contact with a cisgender man (73.8%); these cisgender men might be in sexual networks experiencing the highest prevalence of mpox (*1*). These findings are similar to those from an analysis of mpox cases occurring in 62 transgender women in Europe and the Americas during May–October 2022 (*6*); in that study, the likeliest route of transmission for most transgender women (89%) was sexual contact, with a majority reporting having had a male sexual partner during the preceding month.

Similar to cisgender adults with mpox, Hispanic (37.0%) and Black (27.6%) transgender and gender-diverse persons were disproportionately represented among mpox cases compared with the racial and ethnic percentage distribution of the overall U.S. population.*** Unique health disparities and barriers to prevention and care faced by transgender and gender-diverse persons might be exacerbated by racial and ethnic health disparities (*3*,*4*). Ensuring the prioritization of eligible transgender and gender-diverse persons for mpox vaccination, expanding community engagement and outreach to improve prevention messages, and ensuring equity in approaches to mpox testing, treatment, and prevention strategies are critical public health priorities.

The findings in this report are subject to at least three limitations. First, data on certain variables such as symptoms reported, HIV status, hospitalization status, and exposure information were frequently missing in national case surveillance data. Recent sexual and close intimate contact information was missing for approximately one in five cases in transgender and gender-diverse persons, and among those with available information, approximately one in four did not report recent sexual or close intimate contact with a cisgender man. These missing data limit the ability to fully characterize the epidemiologic and clinical features of transgender and gender-diverse persons with mpox. In-depth collection of accurate exposure information is vital to understanding how persons without recent sexual or close intimate contact with cisgender men are likely being exposed to mpox virus, which could guide expanded prevention messaging. Second, methods for collecting sex and gender information are not standardized across all U.S. states and territories. Self-reported gender or sex assigned at birth were missing for 10,370 (36.9%) adults, and for these persons, gender identity was presumed to be cisgender unless gender identity was reported as transgender or another gender identity. This limitation could have resulted in undercounting persons who identify as transgender or gender-diverse, particularly in jurisdictions that do not routinely collect information about transgender and gender-diverse identities. Finally, in the absence of available data on sex assigned at birth, current sex might have been reported, potentially leading to misclassification of gender identity. Improving collection of data on transgender and gender-diverse persons, such as standardizing approaches to collection of sex and gender information and routinely capturing the diversity of gender identities, is important to better understanding and addressing health inequities.

This analysis found that transgender and gender-diverse adults have been experiencing a disproportionate prevalence of *Monkeypox virus* infections, particularly among those who are Hispanic and Black. Meeting the unique health needs and addressing barriers to prevention and care faced by many transgender and gender-diverse persons is a critical public health priority, particularly during the current mpox outbreak. Adequately addressing the needs of this population will require standardized collection of data on sex and gender identity. Tailoring public health prevention and outreach efforts to transgender and gender-diverse communities, including the prioritization of eligible transgender and gender-diverse persons for mpox vaccination and expanding community engagement efforts, might reduce the disproportionate prevalence of mpox among this population.

**Summary**

**What is already known about this topic?**

Epidemiologic data on and clinical characteristics of monkeypox (mpox) in transgender and gender-diverse persons are limited.

**What is added by this report?**

The ongoing mpox outbreak has disproportionately affected transgender and gender-diverse (i.e., not cisgender or transgender) adults. The most commonly reported potential exposure among transgender and gender-diverse adults with mpox was recent sexual contact with cisgender men; these men might be in sexual networks experiencing the highest mpox incidence.

**What are the implications for public health practice?**

Addressing the unique health needs faced by many transgender and gender-diverse adults is an important public health priority. Tailoring prevention and outreach efforts to transgender and gender-diverse communities might reduce the disproportionate incidence of mpox in this population.

## Footnotes

* https://www.cdc.gov/poxvirus/monkeypox/response/2022/index.html (Accessed November 9, 2022).

† Transgender and gender-diverse persons are those whose gender identity might differ from their assigned sex at birth. This description includes transgender women, transgender men, and gender-diverse persons identifying as another gender (i.e., not transgender or cisgender), such as nonbinary, genderqueer, and gender nonconforming. Using data from CDC’s 2017–2020 Behavior Risk Factor Surveillance System and 2017 and 2019 Youth Risk Behavior Survey, which rely on self-reporting of gender identity, the Williams Institute estimates that 1.3 million transgender and gender-diverse adults live in the United States; however, this percentage might be an underestimate because some persons might have been reluctant to disclose their gender identity because of fear of stigma or other reasons.

§
https://www.cdc.gov/poxvirus/monkeypox/pdf/sCRF-Short-Form.pdf

¶
https://www.cdc.gov/nndss/index.html

** Case report form responses to “Do you currently describe yourself as male, female, or transgender?” include “male,” “female,” “transgender female,” “transgender male,” and “another gender identity.”

†† Thirty-six adults assigned male sex at birth identified as women and were classified as transgender women; 23 adults assigned female sex at birth identified as men and were classified as transgender men. Information on assigned sex at birth or gender identity was available for 98.4% of all adults with mpox. Among 10,370 (36.9%) persons for whom self-reported gender or assigned sex at birth was missing, gender identity was presumed to be cisgender consistent with the available response, unless their gender (if reported) was transgender or “another gender identity.”

§§ 45 C.F.R. part 46.102(l)(2), 21 C.F.R. part 56; 42 U.S.C. Sect. 241(d); 5 U.S.C. Sect. 552a; 44 U.S.C. Sect. 3501 et seq.

¶¶ Persons of Hispanic origin might be of any race but are categorized as Hispanic; all racial groups are non-Hispanic.

*** According to 2021 U.S. Census Bureau estimates, Black and Hispanic persons constitute 13.6% and 18.9% of the U.S. population, respectively. https://www.census.gov/quickfacts/fact/table/US/PST045221 (Accessed November 15, 2022).
